# Profiling salivary miRNA expression levels in Fanconi anemia patients – a pilot study

**DOI:** 10.1007/s10266-023-00834-9

**Published:** 2023-07-17

**Authors:** Kai Dun Tang, José M. Amenábar, Juliana L. Schussel, Cassius Carvalho Torres-Pereira, Carmem Bonfim, Nevenka Dimitrova, Gunter Hartel, Chamindie Punyadeera

**Affiliations:** 1 Faculty of Health, School of Biomedical Sciences, Centre for Biomedical Technology, Queensland University of Technology, Saliva & Liquid Biopsy Translational Laboratory and Translational Research Institute, Griffith University, 46 Don Yong Road, Nathan, Brisbane, QLD Australia; 2 Stomatology Department, Universidade Federal Do Paraná, Curitiba, Brazil; 3 Bone Marrow Transpantation Unit, Hospital de Clínicas, Universidade Federal Do Paraná, Curitiba, Brazil; 4 New York Medical College, Valhalla, NY USA; 5 Statistics Unit, QIMR Berghofer Medical Research Institute, Brisbane, Australia

**Keywords:** Fanconi anemia, Oral squamous cell carcinoma, miRNAs, Saliva, Biomarker

## Abstract

The overarching goal of this study is to predict the risk of developing oral squamous cell carcinoma (OSCC) in Fanconi anemia (FA) patients. We have compared the microRNA (miRNA, miR) expression levels in saliva samples from FA patients (*n* = 50) who are at a low-moderate and/or high risk of developing OSCC to saliva samples from healthy controls (*n* = 16). The miRNA expression levels in saliva samples were quantified using qPCR. We observed that miR-744, miR-150-5P, and miR-146B-5P had the best discriminatory capacity between FA patients and controls, with an area under the curve (AUC) of 94.0%, 92.9% and 85.3%, respectively. Our data suggest that miR-1, miR-146B-5P, miR-150-5P, miR-155-5P, and miR-744 could be used as panel to predict the risk of developing OSCC in FA patients, with a 89.3% sensitivity and a 68.2% specificity (AUC = 81.5%). Our preliminary data support the notion that the expression levels of salivary miRNAs have the potential to predict the risk of developing OSCC in FA patients and in the future may reduce deaths associated with OSCC.

**Supplementary Information:**

The online version contains supplementary material available at 10.1007/s10266-023-00834-9.

## Introduction

Fanconi anemia (FA) is a rare inherited genetic condition characterized by physical abnormalities, bone marrow failure syndrome and a very high risk of developing cancer [1]. It is usually inherited as an autosomal recessive genetic disorder, but X-linked inheritance has also been reported. It is caused by the loss of function of at least one of 23 genes involved with the FA/BRCA pathway [1]. This pathway is involved in the repair of double-stranded DNA breaks and homologous recombination, as well as repair of interstrand DNA cross-linking (ICL) caused by exogenous agents [2, 3]. If the ICLs are left unrepaired, they will promote genotoxic stress, genomic instability and tumorigenesis [4]. Approximately, 20% of FA patients have some types of malignancy, mainly leukemias [5] or oral squamous cell carcinoma (OSCC) [6, 7] as these are the two most common types of solid tumor in FA patients.

MicroRNAs (miRNAs) are non-coding, small RNAs that regulate the gene expression by modulating many cellular processes, such as controlling and modulating immunity, cell proliferation, promoting survival and growth of malignant cells, and cancer metastasis [8, 9]. A single miRNA can bind to many mRNA sequences and each mRNA sequence is a target of many miRNAs. Compelling evidence has demonstrated that the expression of miRNA is commonly dysregulated in multiple disease states, including FA [10–12]. However, there are few studies analyzing miRNAs expression levels in tumor tissues, plasma or serum of FA patients [13, 14]. For instance, miR-1, miR-146B-5P and miR-150-5P have been indicated as novel plasma biomarkers for aplastic anemia, a condition often found in FA patients [15]. Furthermore, low expression levels of miR-155-5P and miR-181C have been reported in FA patients [13, 14].

Salivary analysis has been proposed as an alternative method of choice to blood based analysis for unravelling disease specific biomarkers [16–19]. Saliva is an ideal diagnostic medium for biomarker detection studies in FA patients because of its non-invasiveness and ease of collection so multiple samples can be collected from an individual cost effectively [20–22]. A further advantage of this biofluid is its proximity to the oral cavity, as this region is one of the most common sites for the development of OSCC [23–25]. To the best of our knowledge, there is thus far no study has attempted to investigate the relationship between salivary FA-associated miRNAs and OSCC risk factors in FA patients. The overarching objective of this study was to investigate whether FA-associated salivary miRNAs signature could be used as a biomarker in FA patients to predict the risk of developing OSCC.

## Materials and methods

### Participants

This study was approved by the Ethics Review Board of the Complexo Hospital de Clínicas da Universidade Federal do Paraná (approval number 2.426.777) as well as by the University of Queensland Medical Ethical Institutional Board [HREC No: 2014000679 and 2014000862]; Queensland University of Technology [HREC No: 1400000617 and 1400000641] and by the Princess Alexandra Hospital Ethics Review Board [HREC Number: HREC/12/QPAH/381]. All participants provided written informed consent before saliva sample collection, following the Declaration of Helsinki. FA patients who attended the Bone Marrow Transplantation Unit, at the Hospital de Clínicas from November 2017 to June 2018, participated in the study. The participants were classified into two groups: low-moderate and high risk of developing OSCC, based on the criteria proposed by Furquim et al. [17]. Briefly, high risk group consisted of FA patients who underwent hematopoietic stem-cell transplantation (HSCT) > 5 years before the most recent examination or ≥ 18 years old and have developed oral potentially malignant disorders (OPMD). The low-moderate risk group consisted of patients over 18 years of age without OPMD, or under 18 years of age with OPMD, or without OPMD but HSCT within the last 5 years. We also recruited a control group, and the inclusion criteria included no self-reported illness or history of any oral or systemic disease.

### Saliva sample collection

Participants were asked to refrain from eating, drinking or brushing their teeth for at least one hour prior to saliva sample collection. All samples were collected in the morning between 7:30 am to 11:30 am to minimize diurinal variations [24, 26–28]. In brief, participants were asked to refrain from eating 1-h prior to saliva collection. Participants were asked to tilt their heads down and pool saliva in the mouth for 2–3 min and expectorate (2–5 mL of saliva) into a 50 mL Falcon tube kept on ice. Samples were transported on dry-ice to the laboratory for further processing. Saliva collection, preprocessing and storage was conducted using previously published protocols [24, 26–28]. After collection, all saliva samples were immediately stored on dry ice and transferred to the laboratory for down stream processing.

### miRNA extraction and cDNA synthesis

In order to avoid batch to batch variations, we have isolated miRNA from saliva samples at one time point. miRNA was isolated and enriched by QIAzol lysis reagent (Qiagen) in combination with the NucleoSpin miRNA kit (Macherey–Nagel, Düren, Germany) as previously described [29]. Briefly, 800μL QIAzol lysis reagent was added to 200μL saliva. Then 200μL chloroform was added and the mixture was then centrifuged at 10,000 × g for 10 min at 4 °C. The upper aqueous layer was transferred into a new microcentrifuge tube containing 200 μL ethanol and subsequently transferred into a Nucleospin column for miRNA enrichment as per manufacturer protocol. The quantity of the isolated miRNA was determined using both Qubit® 3.0 Fluorometer (Thermo Fisher Scientific, Waltham, MA, USA) and Nanodrop ND-1,000 spectrophotometer (Thermo Fisher Scientific). OD 260:280 ratios ≥ 1.8 were accepted as pure.cDNA synthesis was carried out via a miScript II RT Kit (Qiagen) according to the manufacturers’ instructions. Briefly, miRNAs were polyadenylated with polymerase A and transcribed into cDNA using oligo-dT primers (in parallel in the same tube). The oligo-dT had a 3′degenerate anchor and a universal tag sequence at the 5′end allowing amplification of mature miRNA through a real-time PCR step (60 min at 37ºC followed by heat inactivation for 5 min at 95ºC). The polyadenylation and universal tag primer ensure that genomic DNA is not amplified. Therefore, DNAase treatment was omitted from the miRNA isolation protocol.

### miRNA qPCR analysis

The qRT-PCR for miRNA quantitation was performed using target-specific miScript Primer Assays (miR-1, Cat. No. MS00008358; miR-146B-5P, Cat. No. MS00003542; miR-150-5P, Cat. No. MS00003577; miR-155-5P, Cat. No. MS00031486; miR-181C-5P, Cat. No. MS00008841; miR-744 Cat. No. MS00010549) and the miScript SYBR Green PCR Kit (Qiagen), according to the manufacturer’s protocol. For the identification of optimal miRNA normalizer in saliva samples a panel of six miScript™ primer PCR assays (SNORD 61, Cat. No. MS00033705; SNORD68, Cat. No. MS00033712; SNORD72, Cat. No. MS00033719; SNORD95, Cat. No. MS00033726; SNORD96A, Cat. No. MS00033733 and miR-489 Cat. No. MS00007700) were used. Briefly, 3 ng cDNA was used as a template across control and FA patients groups for PCR amplification and the thermocycling conditions were as follows: 95 °C for 15 min to activate hot start Taq DNA polymerase and this was followed by 40 cycles of 94 °C for 15 s, 55 °C for 30 s and 70 °C for 30 s and a final melting curve analysis with the following conditions: 95 °C for 15 s, 60 °C for 60 s, and 95 °C for 15 s using QuantStudio™ 7 Flex Real-Time PCR System (Applied Biosystems). A value of 40 was assigned when the threshold cycle (Ct) value was underdetermined and the average Ct value of the reactions in duplicate were determined. The comparative Delta Ct method was adopted and miR-489 was used as a normalizer as published by us previously. In this method, we have subtracted the ct value of target gene form the normalizer (miR 489). If we get a negative delta Ct value, this would mean that the target gene Ct value is higher (lower expression) than the normalizer.

### Statistical analysis

Statistical analyses were performed using JMP Pro version 16 (SAS Institute, Cary, NC, USA) and GraphPad Prism version 8.0 (GraphPad Software Inc., La Jolla, CA, USA). miRNA expression levels of miR-1, miR-146B-5P, miR-150-5P, miR-155-5P, miR-181C-5P and miR-744 in saliva samples were compared between two cohorts (controls and FA patients) using t-tests; and comparison of the three cohorts (controls, low-moderate and high risk FA patients) were compared using one way ANOVA, followed by the Tukey–Kramer HSD multiple comparison procedure [30, 31]. P-values less than 0.05 were considered significant. Correlation between the predictors was calculated using Pearson correlations. Lasso penalized multiple logistic regression [32] was used to identify a panel of miRNA markers for distinguishing between FA patients classified as low-moderate risk of OSCC from those classified as high risk. The Lasso penalty parameter was chosen to minimize the Akaike's Information Criterion [33]. Sensitivity and specificity were calculated for the predictive model, with the cutoff chosen to maximize the Youden’s Index [34]. Confidence intervals were calculated using the score method [35]. Receiver operating characteristic (ROC) curves were generated for all logistic regressions, and the area under the curve (AUC) with 95% confidence intervals presented to indicate the predictive ability of the models. The AUC of the ROC is also known as the concordance or c-statistic [36].

## Results

### Population characteristics

The demographic and clinical characteristics of controls (*n* = 16) and FA patients (*n* = 50) are summarized in Table 1. Controls and FA patients had a mean age of 23 years (range 18–31 years) and 20 years (range 7–37 years), respectively. Gender distribution was approximately even in each group. All FA patients were considered as non-smokers and non-drinkers while; the majority of controls were regarded as non/former smokers and non-drinkers. Nearly all FA patients were single (95%) while marital status was not known for controls. Nearly all controls were identified as Caucasian (88%) compared to only 50% of FA patients. All high-risk FA patients underwent HSCT, compared to only 50% of the low-moderate risk FA patients. Graft vs host disease (GVHD) and OPMD were diagnosed in 6 (12%) and 9 (18%) FA patients, respectively.

**Table 1:** Participants clinical and demographic characteristics

Sociodemographic characteristics	Controls	FA patients who are at low-moderate risk of developing OSCC	FA patients who are at high risk of developing OSCC	FA Patients
	(*n* = 16)	(*n* = 22)	(*n* = 28)	(*n* = 50)
Age (Mean, years, range)	23 (18–31)	16 (7–30)	23 (12–37)	20 (7–37)
*Gender*				
Male	9	9	12	21
Female	7	13	16	29
*Smoking status*				
Non-smoker	4	22	28	50
Former smoker	4	0	0	0
Smoker	4	0	0	0
Unknown	4	0	0	0
*Drinking status*				
Non-drinker	11	22	28	50
Drinker	1	0	0	0
Unknown	4	0	0	O
*Marital status*				
Married		1	1	2
Not Married		21	27	48
*Ethnicity*				
Caucasian	14	11	14	25
Non-Caucasian	2	11	14	25
*Profession*				
student		18	21	39
worker		4	7	11
*OSCC risk factors*				
*HSCT*				
Yes		11	28	39
No		11	0	11
HSCT time (Mean (range)		3 (1–4)	13 (2–30)	10 (1–30)
*GVHD*				
Yes		1	5	6
No		21	23	44
*OPMD*				
Yes		3	6	9
No		19	22	41

### The stability of the miRNA housekeeping gene in saliva samples collected from FA patients and controls

The distributions of the Ct values of the five reference genes from the SNORD family and miR-489 in saliva samples of controls and FA patients are shown in Supplementary Fig. 1. All reference genes from the SNORD family showed statistical differences between controls and FA patients, but the salivary expression levels of miR-489 were quite similar between controls and FA patients. Furthermore, miR-489 showed the highest level of relative expression (mean Ct = 22.56) with the lowest standard deviation (SD) values of 1.11 compared to the other reference genes from the SNORD family (Supplementary Table 1). Previous findings are consistent with our study showing that miR-489 as the best normalizer for miRNA research when using the body fluids [37].

### The expression levels of FA-associated miRNAs in FA patients

**Fig. 1:**
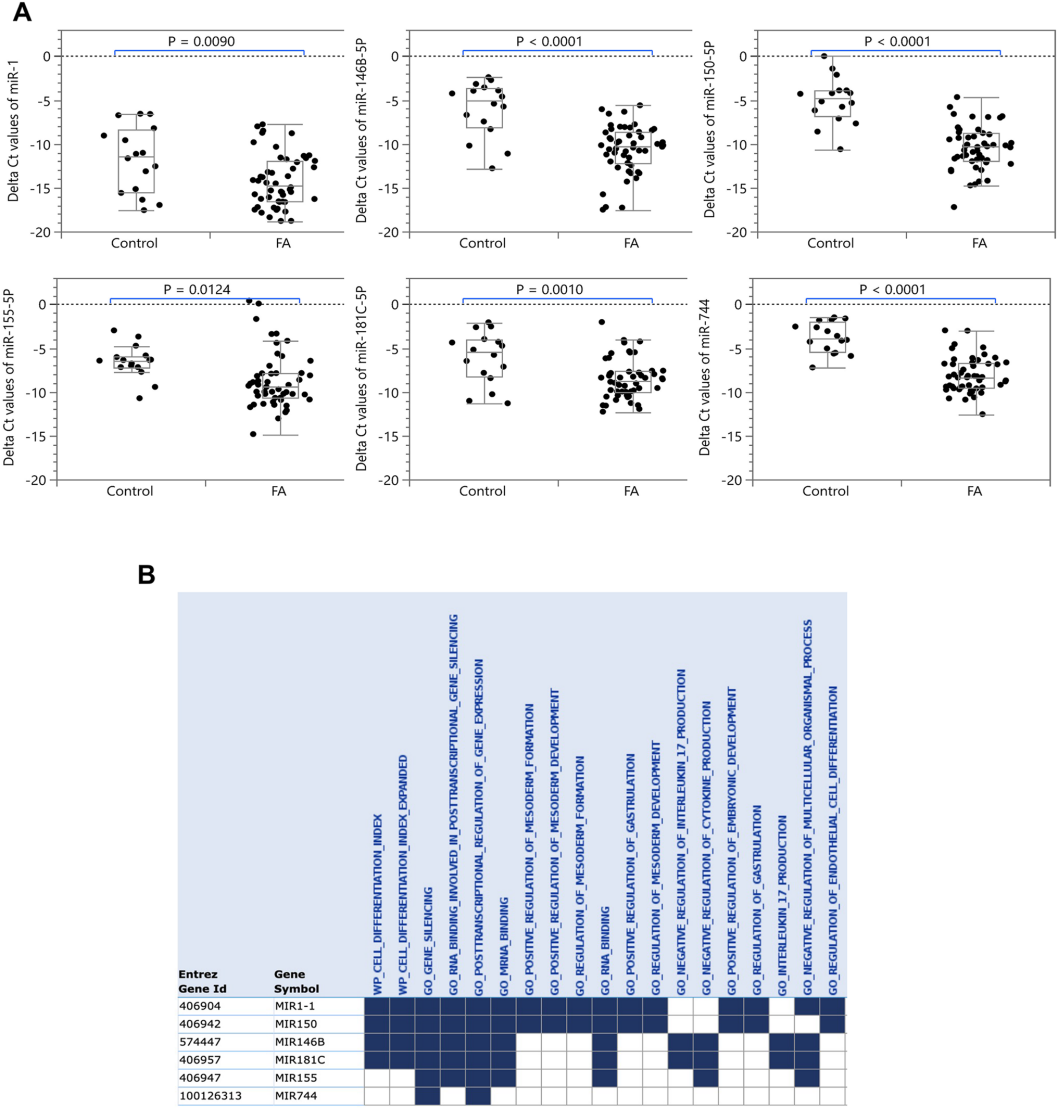
Dysregulation of miRNAs in saliva samples collected from FA patients. **A** The relative expression (ΔCT) of miR-1, miR-146B-5P, miR-150-5P, miR-155-5P, miR-181C-5P and miR-744 in saliva collected from controls and FA patients. **B** The six miRNA statistically significantly overlap with genesets from the MsigDB catalog. Data were normalized with miR-489 and are presented as delta Ct values. Statistically significant differences (P < 0.05) between two groups were determined using Mann–Whitney U-test. (*P* values: * < 0.05, ** < 0.005, *** < 0.001, **** < 0.0001)

Dysregulation of miRNAs has been associated with a number of diseases including FA [13], however their expression levels in saliva samples collected from FA patients still remains to be elucidated. To address this question, we first investigated the expression levels of six literature documented FA-associated miRNAs (miR-1, miR-146B-5P, miR-150-5P, miR-155-5P, miR-181C-5P and miR-744) in saliva samples collected from controls and FA patients. All six FA-associated miRNAs were signficantly downregulated (*P* < 0.05) in saliva samples collected from FA patients compared to controls as shown in Fig. 1A. The diagnostic values of miR‑1, miR-146B-5P, miR-150-5P, miR-155-5P, miR-181C-5P and miR-744 were also investigated using logistic regressions analysis between groups (control *vs* FA) against each of the miRNAs. Each miRNA was statistically significant, however, the best predictors were miR-744, miR-150-5P, and miR-146B-5P (ROC AUC (95% CI) = 94.0% (85.1%, 97.7%), 92.9% (80.7%, 97.6%), and 85.3% (66.8%, 94.3%), respectively, *P* < 0.0001 in each case, Supplementary Fig. 2).

We computed the overlap of these six FA-associated miRNAs with the molecular signature database (MSigDB) which contains 31,117 gene sets [38]. We present in Fig. 1B the 19 statistically significant overlapping gene sets, (FDR q-value ≤ 0.05). In addition to the expected gene sets related to gene silencing and post-transcriptional regulation, we also observed that miR-1 and miR-150-5P are involved in mesoderm development and gastrulation, as well as the regulation of endothelial cell differentiation. Importantly, miR-146B-5P and miR-181C-5P are involved in negative regulation of IL-17.

### Salivary miRNA expression differences in FA patients at low-moderate vs high risk of developing OSCC

**Fig. 2:**
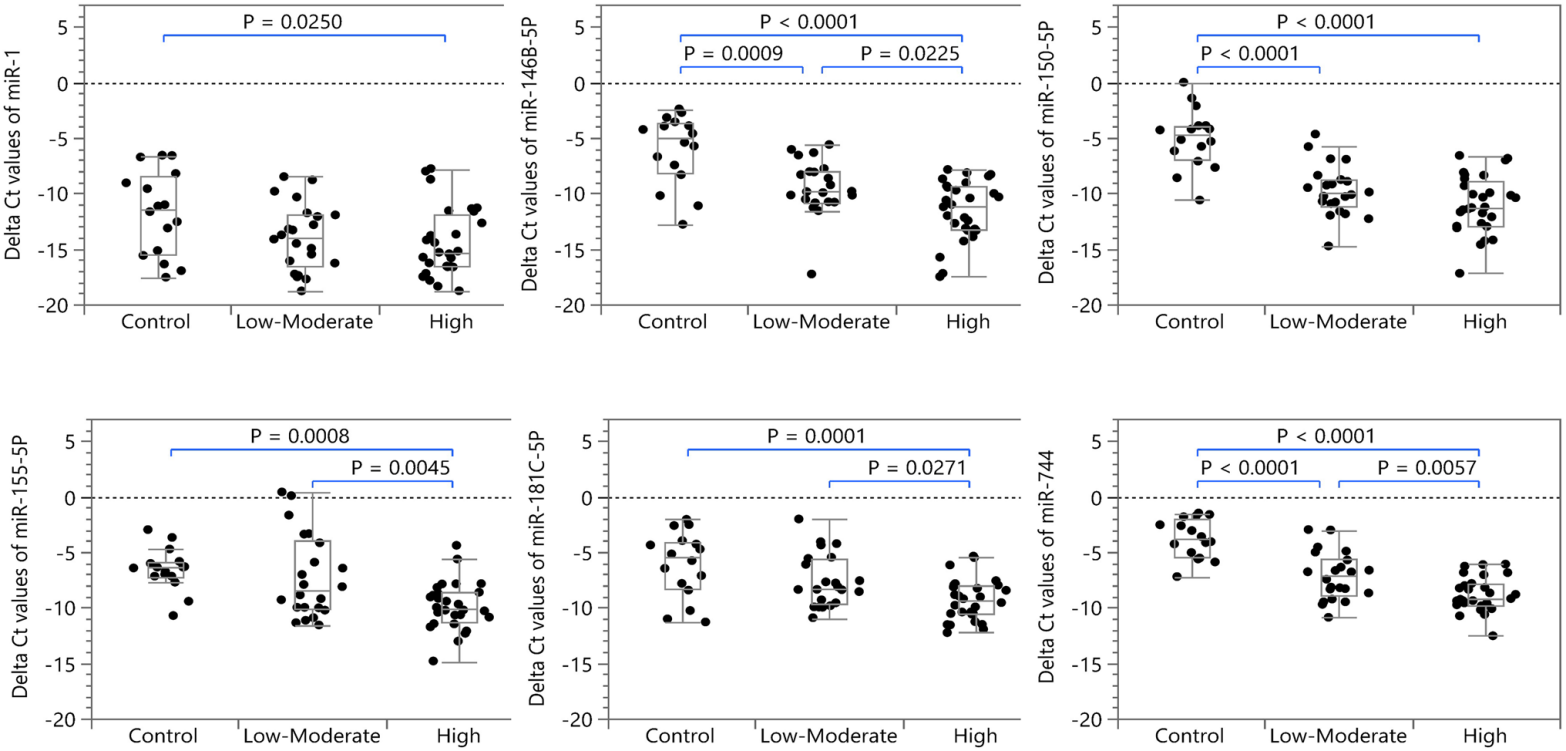
The relative expression of miR-1, miR-146B-5P, miR-150-5P, miR-155-5P, miR181C-5P and miR-744 in saliva collected from controls, low-moderate and high risk of developing OSCC groups. Data were normalized with miR-489 and are presented as delta Ct values. Statistically significant differences (*P* < 0.05) among these three groups were determined using ordinary one way ANOVA. (*P* values: * < 0.05, ** < 0.005, *** < 0.001, **** < 0.0001)

**Fig. 3:**
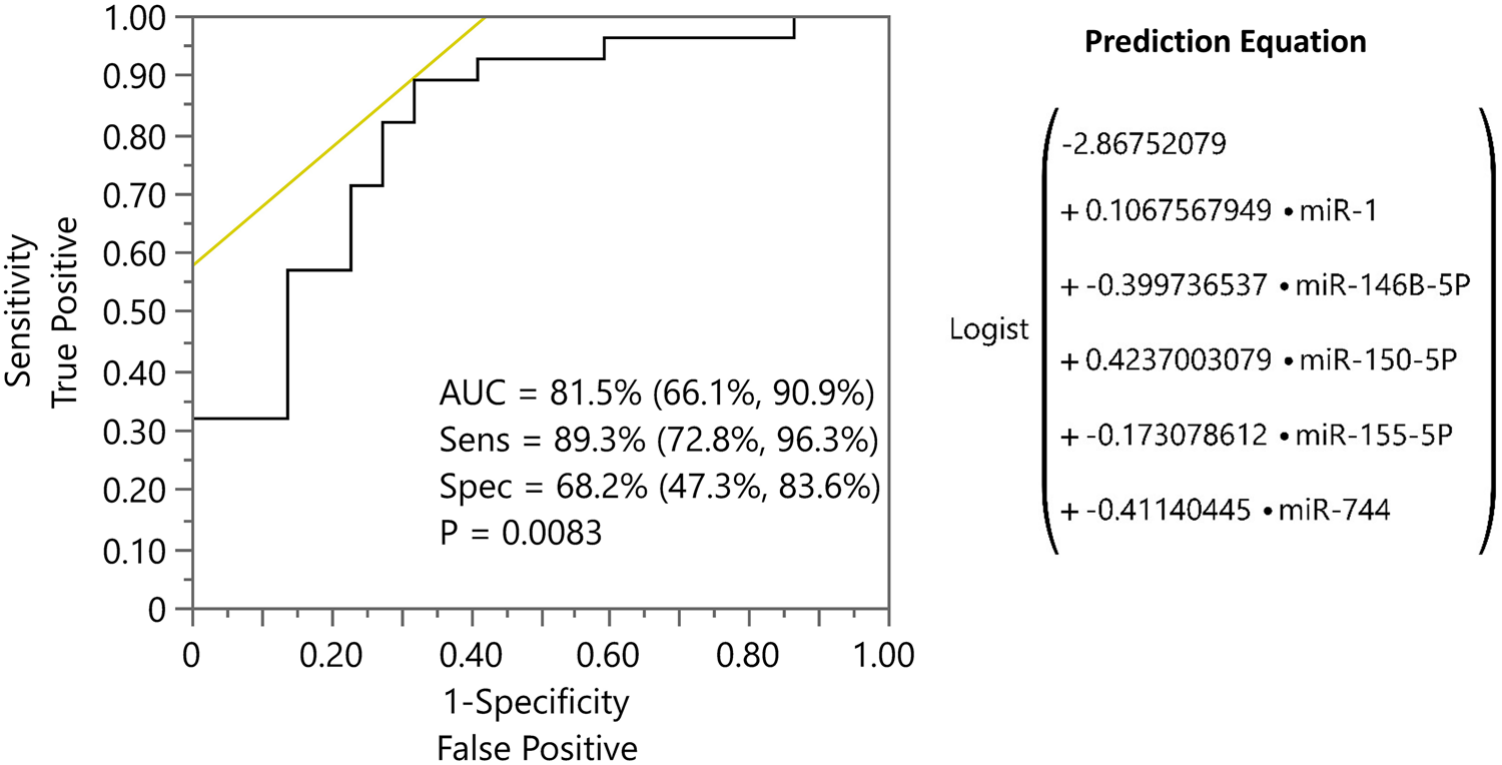
Receiver operator characteristic (ROC) curve combining miR-1, miR-146B-5P, miR-150-5P, miR-155-5P, and miR-744 for predicting FA patients who are at a higher risk of developing OSCC

As shown in Fig. 2, the expression levels of miR-146B-5P, miR-155-5P, miR-181C-5P and miR-744 were significantly downregulated (*P* < 0.05) in saliva samples collected from high risk patients compared to low-moderate risk FA patients. Logistic regressions of OSCC risk levels (low-moderate/high risk) versus individual miRNAs found that miR-146B-5P, miR-155-5P, miR-181C-5P and miR-744 to be significant predictors (*P* < 0.01, in each case), but with relatively low ROC AUCs (< 75%, Supplementary Fig. 3). Furthermore, as the expression levels of the miRNAs are quite strongly correlated (Supplemetary Fig. 4), we used a lasso penalized logistic regression to identify a set of markers that could serve as a potential biomarker panel to discriminate among controls, low-moderate and high risk groups. The model selected miR-1, miR-146B-5P, miR-150-5P, miR-155-5P, and miR-744 as predictors, yielding an ROC AUC = 81.5% (95% CI 66.1%, 90.9%), sensitivity of 89.3% and specificity of 68.2%, based on using Youden’s J statistic to select the cutoff (Fig. 3).

## Discussion

FA is a rare and a complex tumor-prone disease defined by an interlaced genotype and phenotype. Accumulating evidence supports the notions that salivary miRNAs expression changes can be used as potential biomarkers for the detection of oral and systemic diseases [8, 10, 39–41]. However, there is a dearth of available studies investigating the utility of salivary miRNAs as useful biomarkers to identify FA patients who are likely to develop OSCC. Studies have shown that early detection of OSCC improves five-year survival rates from 20 to 80% [42]. As such, it then becomes important to early identify FA patients who are at low-moderate vs high risk of developing OSCC. For the first time, we demonstrate that by combining miR-1, miR-146B-5p, miR-150-5p, miR-155-5P, and miR-744 as a biomarker panel can predict FA patients who are at a higher risk of developing OSCC (AUC = 81.5%, Sensitivity 89.3% and Specificity 68.2%).

In our study, miR-1 and miR-146B-5P were under expressed in the saliva samples from high-risk FA patient group. This is further supported by previous studies demonstrating that the downregulation of both miRNAs is associated with aggressive OSCC [43, 44]. Previous studies have demonstrated that the dysregulation of miR-1 and miR-146B-5P to be associated with the development of aplastic anemia, a common hematological problem in FA patients [15]. Furthermore, the alterations of miR-1 and miR-146B-5P levels in serum have been linked to chronic inflammation [45, 46]. While miR-1 targets B-cell lymphoma 2 (BCL2), miR-146B-5P targets and represses TNF-receptor associated factor 6 (TRAF6) and Interleukin 1 receptor-associated kinase 1 (IRAK1) and has shown to be involved in feedback regulation of the innate immune response.miR-150-5P is mainly expressed in the megakaryocytic lineage, driving megakaryocyte-erythrocyte progenitor differentiation towards megakaryocytes [47]. Previous research has reported that miR-150-5P was expressed at a higher level in plasma of patients with aplastic anemia compared to controls [15]. Conversely, we observed a lower expression level of miR-150-5P in the saliva samples of high-risk FA patients compared to controls. In fact, miR-150-5P was reported to be silenced in OSCC tissues, further supporting the potential of miR-150-5P as a biomarker to predict the FA patients who are at a greater risk of developing OSCC [48].

We found significantly lower levels of miR-155-5P in saliva samples from high-risk FA patients. Similarly, a study by Degan et al. reported lower expression of miR-155-5P in blood samples of FA patients compared to healthy controls [13]. Furthermore, the under expression of miR-155-5P was associated with an increased risk of OSCC metastasis development [49]. Earlier studies have identified miR-155-5P as a critical regulator of inflammation as well as regulating the innate and adaptive immune responses [50]. Several studies have demonstrated that miR-155-5P is required for normal functions of B and T lymphocytes and is up-regulated upon B and T cell activation [51, 52]. Since FA is characterized by chromosomal breakage, miR-155-5P downregulation is obvious.

We found in our study that miR-181C-5P levels to be significantly lower in high-risk FA group compared to both control and low-moderate risk FA groups. Similarly, a study has shown significantly lower levels of miR-181C in saliva samples collected from patients with oral low-grade dysplasia (LGD) who are at a higher risk of developing OSCC compared to those patients with non-progressive oral LGD [53]. This then suggests that miR-181C levels in saliva samples may be used as a non-invasive biomarker to predict OSCC in OPMD patients. It is also reported that miR-181C is downregulated in lymphoblastoid cell lines and fresh peripheral blood cells from FA patients. Also, miR-181C has shown to play an important part in regulating the FA hematopoietic progenitors by targeting the TNFα [14].

Several studies have reported the role of miR-744 in regulating the cellular processes which may eventually contribute to the development of human diseases [54, 55]. Moreover, miR-744 could function as a tumor suppressor or oncogene and the role vary based on the cancer types. In laryngeal squamous cell carcinoma, miR-744 functions as an oncogene and has been demonstrated to be associated with cancer metastasis [56], while miR-744 expression is downregulated in non-small lung cancer [57]. However, little attention has been paid to investigate the role of miR-744 in oral carcinogenesis. We found significantly lower expression levels of miR-744 in the saliva samples from high vs low-moderate risk FA patients and in saliva from controls. Strikingly, one of the potential targets of miR-744 is FANCG [58], which is one of the most likely affected genes in FA. This is a pilot study with some limitations: the modest sample size and a single site recruitment for controls and FA patients, respectively. It would have been appropriate to also include an OSCC group and investigate the miRNA expression changes of established biomarkers of OSCC such as miRNA-21, miR-9 [59]. However, because of the age at with OSCC occurs (55 years), it was not possible to recruit age matched controls and FA patients. Future studies using a large group of FA patients from diverse geographic regions are warranted to investigate the potential clinical utility of salivary miRNAs biomarkers panel in FA patients.

## Conclusions

In conclusion, all of the miRNAs analyzed in this study are downregulated in saliva samples of FA patients when compared with controls. Additionally, miR-1, miR-146B-5P, miR-150-5P, miR-155-5P, and miR-744 have the potential to distinguish high and low-moderate risk of developing OSCC. Our data is promising to predict the risk of developing OSCC in FA patients, using non-invasive miRNA based biomarkers. However, future trial should focus on addressing the current short-comings.

### Supplementary Information

Below is the link to the electronic supplementary material.

Supplementary file1 (DOCX 706 KB)

## Data Availability

All datasets generated for this study are included in the article.
