# Supplementary material for: Profiling salivary miRNA expression levels in Fanconi anemia patients – a pilot study
Source: Odontology. 2023 Jul 17;112(1):299–308. doi: 10.1007/s10266-023-00834-9 (PMC10776736; doi:10.1007/s10266-023-00834-9)
Supplement: Supplementary file 1 — Supplementary file1 (DOCX 706 KB) [file 10266_2023_834_MOESM1_ESM.docx]

**Supplementary Table 1: Housekeeping miRNAs**

| **Housekeeping miRNAs** | **SNORD61** | **SNORD68** | **SNORD72** | **SNORD 95** | **SNORD96A** | **miR-489** |
| --- | --- | --- | --- | --- | --- | --- |
|  |  |  |  |  |  |  |
| Minimum | 21.61 | 24.47 | 30.15 | 20.55 | 21.44 | 20.27 |
| 25% Percentile | 29.22 | 31.69 | 33.70 | 24.86 | 26.20 | 22.20 |
| Median | 31.77 | 36.50 | 40.00 | 29.64 | 32.40 | 22.58 |
| 75% Percentile | 32.90 | 40.00 | 40.00 | 31.86 | 33.73 | 23.21 |
| Maximum | 34.28 | 40.00 | 40.00 | 40.00 | 40.00 | 24.75 |
| Range | 12.67 | 15.53 | 9.85 | 19.45 | 18.57 | 4.48 |
|  |  |  |  |  |  |  |
| Mean | 30.57 | 35.36 | 36.91 | 29.00 | 30.85 | 22.56 |
| Std. Deviation | 3.20 | 4.99 | 3.69 | 4.47 | 4.58 | 1.11 |
| Std. Error of Mean | 0.73 | 1.15 | 0.85 | 1.03 | 1.05 | 0.26 |

**Supplementary Figure 1:** Establishment of an optimal housekeeping gene for salivary miRNA analysis in FA patients.

Data are presented as the Ct values. Statistically significant differences (P < 0.05) between controls (Black) and FA patients (Grey) were determined using Mann-Whitney U-test. (P values: *** < 0.001 and **** < 0.0001).


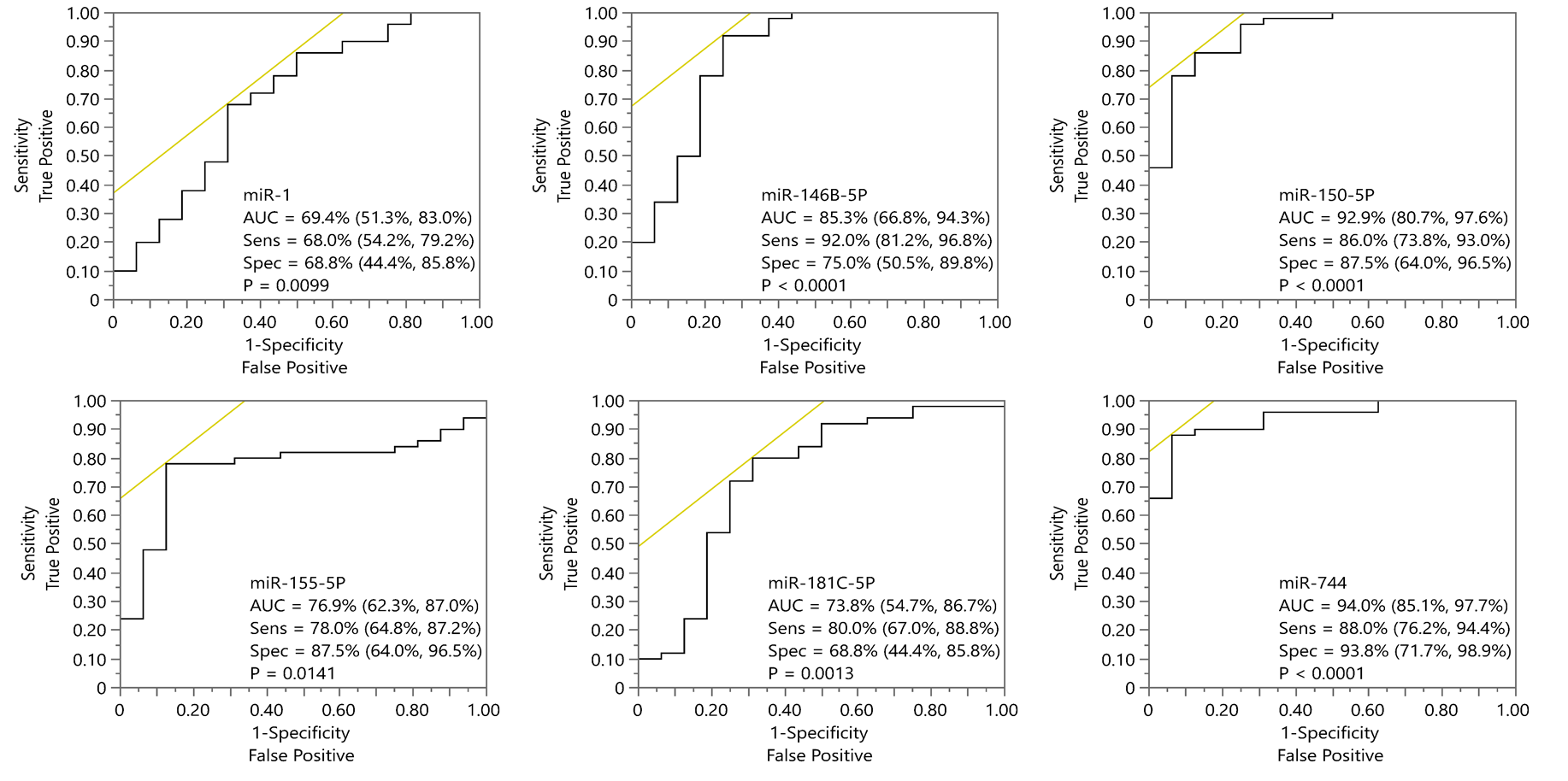


**Supplementary Figure 2:** The receiver operator characteristic (ROC) curve analysis of miRNAs in disciminating between controls and FA patients.

**
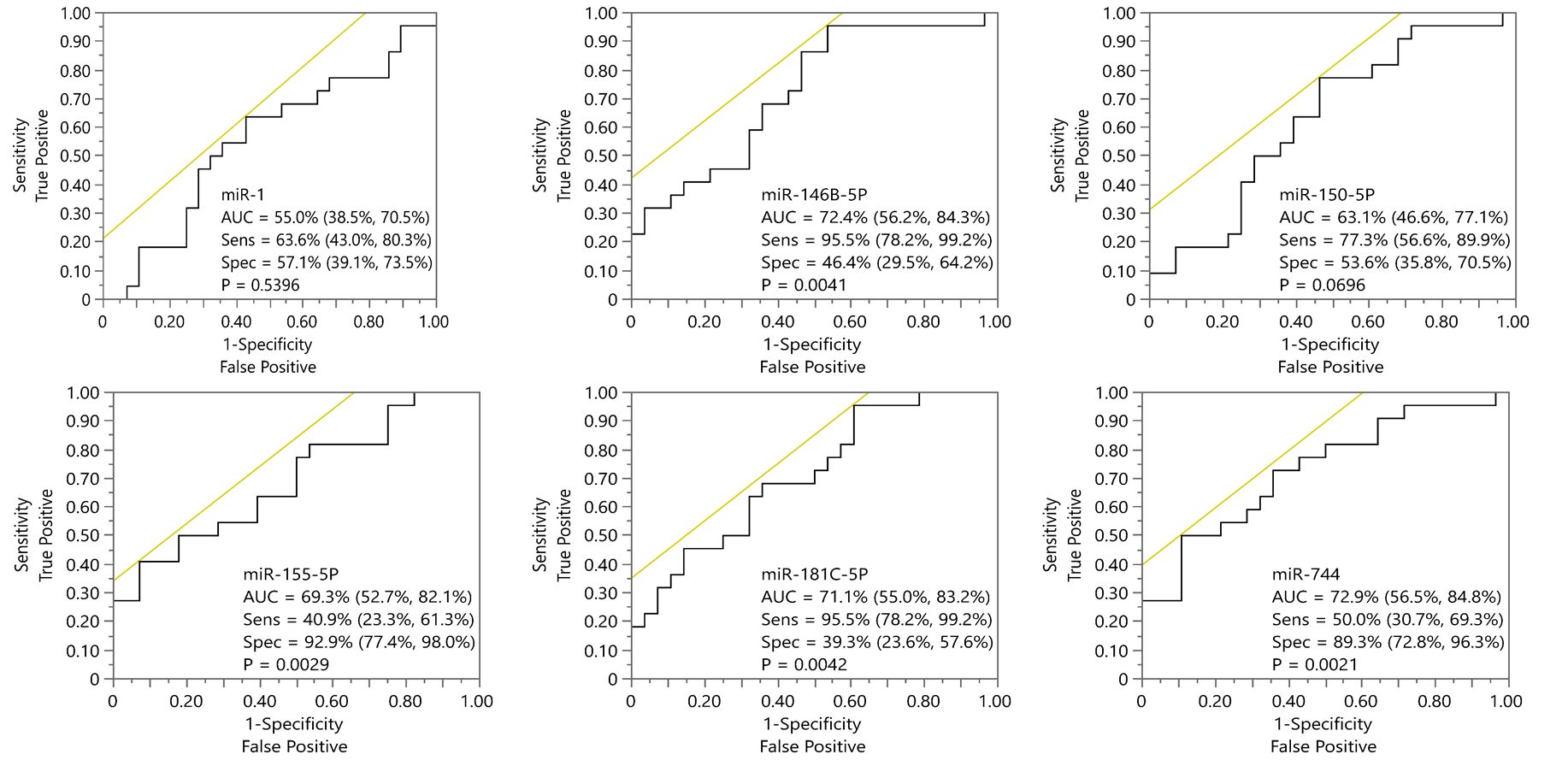
**

**Supplementary Figure 3:** The receiver operator characteristic (ROC) curve analysis of miRNAs in disciminating between low-moderate and high risk groups.


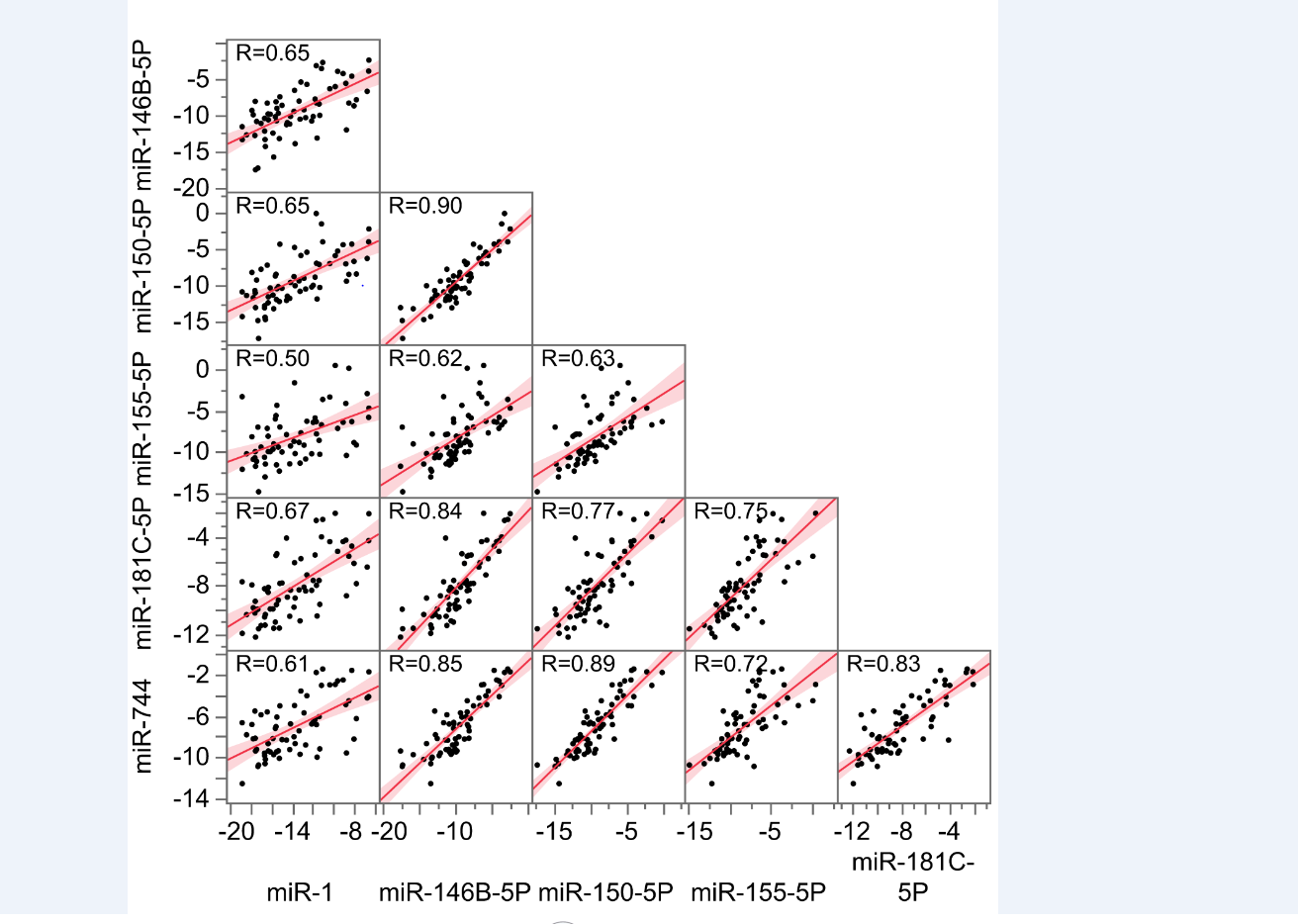


**Supplementary Figure 4:** Correlation among miRNAs. (**P values* < 0.0001)
